# An Antimicrobial Peptidomimetic Induces Mucorales Cell Death through Mitochondria-Mediated Apoptosis

**DOI:** 10.1371/journal.pone.0076981

**Published:** 2013-10-03

**Authors:** E. Magda Barbu, Fazal Shirazi, Danielle M. McGrath, Nathaniel Albert, Richard L. Sidman, Renata Pasqualini, Wadih Arap, Dimitrios P. Kontoyiannis

**Affiliations:** 1 David H. Koch Center, Department of Genitourinary Medical Oncology, the University of Texas M. D. Anderson Cancer Center, Houston, Texas, United States of America; 2 Department of Infectious Diseases, the University of Texas M. D. Anderson Cancer Center, Houston, Texas, United States of America; 3 Harvard Medical School, Boston, Massachusetts, United States of America; 4 Department of Neurology, Beth Israel Deaconess Medical Center, Boston, Massachusetts, United States of America; Geisel School of Medicine at Dartmouth, United States of America

## Abstract

The incidence of mucormycosis has dramatically increased in immunocompromised patients. Moreover, the array of cellular targets whose inhibition results in fungal cell death is rather limited. Mitochondria have been mechanistically identified as central regulators of detoxification and virulence in fungi. Our group has previously designed and developed a proteolytically-resistant peptidomimetic motif _D_(KLAKLAK)_2_ with pleiotropic action ranging from targeted (i.e., ligand-directed) activity against cancer and obesity to non-targeted activity against antibiotic resistant gram-negative rods. Here we evaluated whether this non-targeted peptidomimetic motif is active against Mucorales. We show that _D_(KLAKLAK)_2_ has marked fungicidal action, inhibits germination, and reduces hyphal viability. We have also observed cellular changes characteristic of apoptosis in _D_(KLAKLAK)_2_-treated Mucorales cells. Moreover, the fungicidal activity was directly correlated with vacuolar injury, mitochondrial swelling and mitochondrial membrane depolarization, intracellular reactive oxygen species accumulation (ROS), and increased caspase-like enzymatic activity. Finally, these apoptotic features were prevented by the addition of the ROS scavenger *N*-acetyl-cysteine indicating mechanistic pathway specificity. Together, these findings indicate that _D_(KLAKLAK)_2_ makes Mucorales exquisitely susceptible via mitochondrial injury-induced apoptosis. This prototype may serve as a candidate drug for the development of translational applications against mucormycosis and perhaps other fungal infections.

## Introduction

Mucormycosis, infection caused by the Mucorales fungi, is second in frequency only to aspergillosis, and affects mainly severely immunocompromised individuals, especially those with malignancies and hematologic stem-cell or solid-organ transplants [1,2]. In this critically-ill patient population, the mortality rates exceed 50% when brain or lungs are the major targets and 90% with disseminated infections [1-3]. Mucorales are notoriously resistant to most antifungals, except to toxic agents such as polyene amphotericin B (AMB) and triazole posaconazole, and even these agents are relatively inefficient unless administered very early in the invasive stage, when fungal infection is often very difficult to recognize [4,5]. The genomic sequencing of the most common cause of invasive mucormycosis, *Rhizopus oryzae*, has suggested that Mucorales innate resistance to therapy is the result of a duplication within the genome of ergosterol biosynthesis pathway genes and mitochondrial protein complexes associates with respiratory electron transport chains [6]. Hence, development of new antifungal agents with distinct mechanisms-of-action against mucormycosis, especially those targeting the above mentioned pathways, clearly remains a major unmet need of contemporary medicine.

Antimicrobial peptides, a first line of defense of all species, have been long considered excellent models for antibiotic and antifungal drug development [7]. This vast group of molecules (>1,200) targets a wide spectrum of pathogens and demonstrates effective neutralizing activity [7]. Although most of these peptides presumably act through cell membrane permeabilization, recent reports have emphasized additional functions, including immunomodulatory and wound-healing activities [8]. Notably, peptides displaying selective toxicity against fungi have also been reported: a recent example is the lipopeptide echinocandins, that inhibit the activity of 1,3 β-glucan synthase, an enzyme vital for fungal wall synthesis [9]; however, despite their tentatively broad and specific mechanism of action, these peptides have no activity against Mucorales species [9].

Despite the potential of antimicrobial peptides as drugs, translational development has been hindered mainly by their poor pharmacokinetic attributes, especially their rapid proteolytic degradation along with high manufacturing costs [10]. Attempts to engineer more effective synthetic mimics have focused on the substitution of non-natural D- or β-amino acid residues for the normal L-residues, a process that renders peptidomimetics resistant to proteolysis [11-13]. Studies from our group and others have established that peptide-targeted (i.e., ligand-directed) drugs containing the all-D-enantiomer, _D_(KLAKLAK)_2_, are effective therapeutic candidates for cancer and obesity [14-22]. We have also recently shown that the non-targeted version of this peptidomimetic per se maintains its value as an antimicrobial and retains its membrane-disrupting activity against Gram-negative bacteria, independently of bacterial growth stage or preexisting antibiotic resistance [23].

In the work presented here, we show that _D_(KLAKLAK)_2_: (i) is strongly active against Mucorales, (ii) induces cell and mitochondrial membrane disruption and depolarization, and (iii) triggers fungal cell death specifically through a reversible apoptotic mechanistic pathway. Collectively, our data indicate that _D_(KLAKLAK)_2_ has great potential for development of translational applications *in vivo* against mucormycosis, a frequent and inherently challenging infection in critical patients.

## Materials and Methods

### Drugs and peptidomimetics

AMB (5 mg/ml; Sigma), FLU (2 mg/ml; Sigma), streptomycin (50 mg/ml; Sigma), colistin sulfate (15 mg/ml; Sigma), _D_(KLAKLAK)_2 ,_ and _D_(CVRAC) (100 mg/ml; PolyPeptide Laboratories) were commercially obtained and prepared in sterile water with aliquots stored at -20°C until use. AMB served as a positive control at one-half MIC (2 µg/ml) or MIC (4 µg/ml) [24], FLU and _D_(CVRAC) served as negative controls at 128 µg/ml and 300 µg/ml, respectively. Colistin served as a positive control for the ATP efflux assay at 32 µg/ml [24].

### Isolates and growth conditions

Clinical isolates were grown on yeast extract agar glucose (YAG) plates. After 48 hours at 37°C, spores were collected in sterile saline containing 0.08% Tween-20, washed twice in saline, filtered and enumerated in a hemocytometer. Spores were stored at 4°C in phosphate-buffered saline (PBS) containing streptomycin (100 µg/ml). Spores where grown to germlings or mycelia in RPMI 1640 buffered with MOPS (3-[N- morpholino] propanesulfonic acid) at a final concentration of 0.165 mol/L at pH 7.0 with glutamine and without bicarbonate.

### Susceptibility testing

Broth microdilution was performed as recommended by the Clinical and Laboratory Standards Institute (CLSI) guidelines [25]. To determine the minimum fungicidal concentration (MFC), an aliquot (20 µl) taken from each well that showed 100% growth inhibition and from the last well showing growth similar to that in the control well were plated onto YAG plates. After 24 hours incubation at 37°C, the MFC was registered as the lowest drug concentration at which no growth was observed.

### Germination assay

To determine whether _D_(KLAKLAK)_2_ affects spore germination, we suspended spores (10^5^/ml) in drug-containing RPMI 1640. After six hours, an aliquot (1 ml) was removed from the culture. Organisms were collected by centrifugation at 13,000 x g for five min, washed one time in PBS and fixed in 100 µl of PBS containing 4% paraformaldehyde. The formation of germlings was determined by bright field microscopy (Olympus IX-70; Olympus, Melville, NY) at 400-fold magnification [24].

### Post-antifungal effect

To determine the delay in logarithmic growth upon exposure to _D_(KLAKLAK)_2_, we exposed spores (10^6^/ml) to drug-containing RPMI 1640 for one hour, washed three times in PBS and re-suspended in drug-free RPMI 1640. The logarithmic growth in RPMI 1640 was subsequently determined by measuring the OD_405 nm_ every 20 min for the first hour of incubation at 37°C and every hour afterwards. The post-antifungal effect interval was calculated as the difference between the lag time of each drug concentration and the lag time of the free-drug well [26].

### Viability assay

To assess the fungicidal effect of _D_(KLAKLAK)_2,_ spores (10^4^/ml) were grown to mycelia in microcentrifuge tubes with RPMI 1640 containing 0.15% (wt/vol) Junlon (Nihon Junyaku, Tokyo, Japan) at 37°C with shaking for 18 hours. Medium was removed by centrifugation at 13,000 x g and mycelia were re-suspended in RPMI 1640 containing test drugs for 6 hours. Next, mycelia were washed twice in 0.1 M 3-(N-morpholino) propanesulfonic acid, pH 7 (MOPS buffer) to remove drugs, and incubated with bis-(1,3-dibutylbarbituric acid) trimethine oxonol (DiBAC; Molecular Probes) at 2 µg/ml final concentration, as described [24]. After one hour, samples were washed twice in MOPS buffer and mycelia were mounted on glass slides. Images were acquired by using a fluorescent microscope (Olympus BX-71; Olympus, Melville, NY) with a fluorescein isothiocyanate (FITC) filter at 400-fold magnification.

### XTT reduction assay

We measured the extent of hyphal damage over time upon exposure to _D_(KLAKLAK)_2_ with the 2,3-bis[2-methyloxy-4-nitro-5-[(sulfenylamino) carbonyl]-2*H*-tetrazolium-5-carboxanilide] (XTT) formazan reduction assay as described [27,28]. To obtain mycelia, *R. oryzae* and *M. circinelloides* spores (10^4^/ml) were suspended in RPMI 1640, dispensed into 96-well microtiter plates (100 µl/well) and incubated at 37°C for 18 hours. Drugs diluted in RPMI 1640 were then added to the wells (100 µl/well), and incubated at 37°C. Drug-free RPMI 1640 served as the control medium. After 0, 2, 4, 6, or 24 hours, 1 mg of XTT and 0.17 mg of menadione (Sigma) were added to each well. Plates were incubated at 37°C for an additional hour, and absorbance was measured at OD_450 nm_. Hyphal viability for each time point and drug concentration was calculated as percent of the control well (set to a value of 100%).

### ATP release assay

We assessed the severity of _D_(KLAKLAK)_2_-induced membrane damage by amount of cellular ATP released into the medium. *R. oryzae* or *M. circinelloides* spores were enumerated in a hemacytometer and suspended in RPMI 1640 at 10^7^ cells/ml. After six hours of incubation at 37°C, the medium was removed by centrifugation at 13,000 x g for five min and germlings were re-suspended in drug-containing or drug-free RPMI 1640. After 5, 30, 60, and 90 min of incubation, germlings were removed by centrifugation as described above and the ATP released in the supernatants was assayed by using the CellTiter-Glo luminescent kit (Promega). Data were recorded with a microplate luminometer (Spectramax M5; Molecular Devices) [25,29].

### Transmission electron microscopy

*R. oryzae* spores (10^6^ /ml) were grown for five hours at 37°C. After the generation of germlings was observed by bright field microscopy, drugs were added to the cultures and incubated at 37°C for an additional hour. The ultrastructural changes in germlings features induced by the presence of drugs compared to antifungal-free controls were assessed by TEM at 6,000-fold as described [24].

### FM4-64 staining

Vacuolar morphological changes were visualized by staining with the lipophilic styryl dye *N*-(3-triethylammoniumpropyl)-4-(*p*-diethylaminophenylhexatrienyl)pyridinium dibromide (FM4-64) (Invitrogen). *R. oryzae* hyphae were generated as described above and incubated with FM4-64 at a final concentration of 5 µM for 30 min at RT. After removing the excess dye, hyphae were re-suspended in drug-containing RPMI 1640 and incubated for 60 min at 37°C with shaking. Hyphae were collected, washed three times in PBS and mounted on glass slides. Images were acquired under a triple-band fluorescent microscope (Olympus BX-71; Olympus, Melville, NY) with a rhodamine filter at 400-fold magnification [30].

### MitoTracker staining

Mitochondria swelling was observed by staining with 2-[3-[5,6-dichloro-1,3-bis[[4-(chloromethyl)phenyl]methyl]-1,3-dihydro-2H-benzimidazol-2-ylidene]-1-propenyl]-3-methyl-, Benzoxazolium chloride (MitoTracker Green FM) (Invitrogen). *R. oryzae* spores were cultivated in RPMI 1640 at 37C°. After six hours, the germlings were harvested and resuspended in medium containing 200 nM MitoTracker for 30 min at 37C°. The excess dye was removed by washing three times with 50 mM phosphate buffer, pH 6.0. Images of fixed germlings in paraformaldehyde (4%) were acquired under the FITC filter at 400-fold magnification [31].

### Detection of intracellular reactive oxygen species

Intracellular ROS levels in *R. oryzae* and *M. circinelloides* germlings were measured as described [32]. Germlings were treated with 150 µg/ml of _D_(KLAKLAK)_2_ for three hours at 37 °C, and then spiked with DHR-123 (5 µg/ml). After two hours at RT, the germlings were harvested at 13,000 x g for five min and observed with a Nikon Microphot SA fluorescence microscope (excitation, 490 nm; emission, 590 nm). For quantitative assays, fluorescence intensity values were recorded by using a POLARstar Galaxy microplate reader (excitation, 488 nm; emission, 525 nm; BMG LABTECH, Offenburg, Germany). N-acetyl cysteine (NAC) was used as a ROS scavenger at a final concentration of 40 mM.

### Mitochondrial membrane potential (ΔΨ_m_) measurements

Mitochondrial membrane depolarization was assessed by staining with RH-123, a fluorescent dye that distributes in the matrix in response to electric potential as described [32,33]. Briefly, germlings exposed to drugs for three hours at 37 °C were harvested via centrifugation, washed twice, and re-suspended in PBS. RH-123 was added to the final concentration of 10 µM, and then the mixture was incubated for 30 min in the dark at RT. NAC was used as a ROS scavenger at 40 mM final concentration. Fluorescence intensity was measured as described above.

### Cytochrome *c* release from mitochondria

cyt *c* release into the cytosol was performed as described [32,34]. To isolate mitochondria, *R. oryzae* and *M. circinelloides* germlings were allowed to grow for five hours at 37 °C. Germlings were then harvested and resuspended in fresh RPMI broth containing either 150 µg/ml of _D_(KLAKLAK)_2_ or 2 µg/ml of AMB for three hours at 37 °C. Cells were harvested by centrifugation at 5,000 × *g* for five min and the pellet was homogenized in a 50 mM Tris, pH 7.5, 2 mM ethylenediaminetetraacetic acid (EDTA), 1 mM phenylmethylsulfonyl fluoride. Next, the admixture was supplemented with 2% glucose and centrifuged at 2,000 x *g* for 10 min to remove cellular debris and unbroken cells. The supernatant was collected, centrifuged at 30,000 × *g* for 45 min and then used to estimate cyt *c* in cytoplasm. To obtain pure mitochondria, the pellet was washed in 50 mM Tris (pH 5.0) and 2 mM EDTA, incubated for five min, and centrifuged at 5,000 x *g* for 30 seconds. Mitochondria were suspended in 2 mg/ml of Tris-EDTA buffer. After being reduced by 500 mg/ml ascorbic acid at RT for five min, the amount of cyt *c* in the cytosolic and mitochondrial fractions was measured at 550 nm with a POLARstar Omega spectrophotometer (BMG LABTECH, Ortenburg, Germany).

### Detection of metacaspase activity

Activation of metacaspases was detected with the CaspACE FITC-VAD-FMK In Situ Marker [35]. Germlings were pretreated with drugs for three hours at 37°C. Cells were harvested, washed in PBS, and then re-suspended in 10 µM CaspACE FITC-VAD-FMK solution. After two hours of incubation at 30°C, germlings were washed twice and re-suspended in PBS. Samples were mounted and viewed in a Nikon fluorescence microscope (emission, 488 nm; excitation, 520nm).

### Statistical analysis

For all assays, three independent experiments were carried out in triplicates. Comparisons of multiple treatment groups were performed by using two-way analysis of variance with post-hoc paired comparisons by Dunnett’s test.

Calculations were made with InStat (GraphPad Software). Two-tailed P values of less than 0.05 were considered statistically significant.

## Results and Discussion

### Mucorales are susceptible to _D_(KLAKLAK)_2_

In previous work, we demonstrated that _D_(KLAKLAK)_2_, a proteolytically-resistant peptide-like motif, is active against several Gram-negative rods, irrespective of their pre-existent antibiotic resistance [23]. Cellular and model membrane assays revealed that this peptidomimetic disrupts the lipid bilayer in a detergent-like manner, resulting in death of bacteria due to dissipation of proton-motive force [23]. Therefore, we hypothesized that _D_(KLAKLAK)_2_ may have similar effect on fungal membranes. Susceptibility testing by broth microdilution [25] in RPMI 1640 revealed complete growth inhibition of Mucorales clinical isolates (Table 1). The average minimum inhibitory concentration (MIC) was 300 µg/ml and the minimum fungicidal concentration (MFC) was twice the MIC (median MFC/MIC ratio, 2) (Table 1). When yeast extract-agar-glucose medium (YAG) was used, similar values were recorded for *R. oryzae*. However, the MICs against *Mucor circinelloides* were higher (~600 µg/ml) and an MFC value could not be determined in these growth conditions (Table 1).

**Table 1:** Minimum inhibitory concentration (MIC) and minimum fungicidal concentration (MFC) for *Mucorales* isolates (µg/ml).

**Isolate**	**Test medium**			
	**RPMI 1640**		**YAG**	
	**MIC**	**MFC**	**MIC**	**MFC**
*Mucor circinelloides* 4030	300	600	600	Not active
*Mucor circinelloides* 4480	300	600	600	Not active
*Mucor circinelloides* 5904	300	600	600	Not active
*Rhizopus oryzae* 3140	300	600	300	Not active
*Rhizopus oryzae* 4153	300	600	300	Not active
*Rhizopus* sp. 4523	300	600	300	Not active
*Rhizopus homothallicus* 5790	300	600	300	Not active
*Rhizopus oryzae* 5799	300	600	300	Not active
*Rhizopus oryzae* 6093	300	600	300	Not active

**Figure 1:**
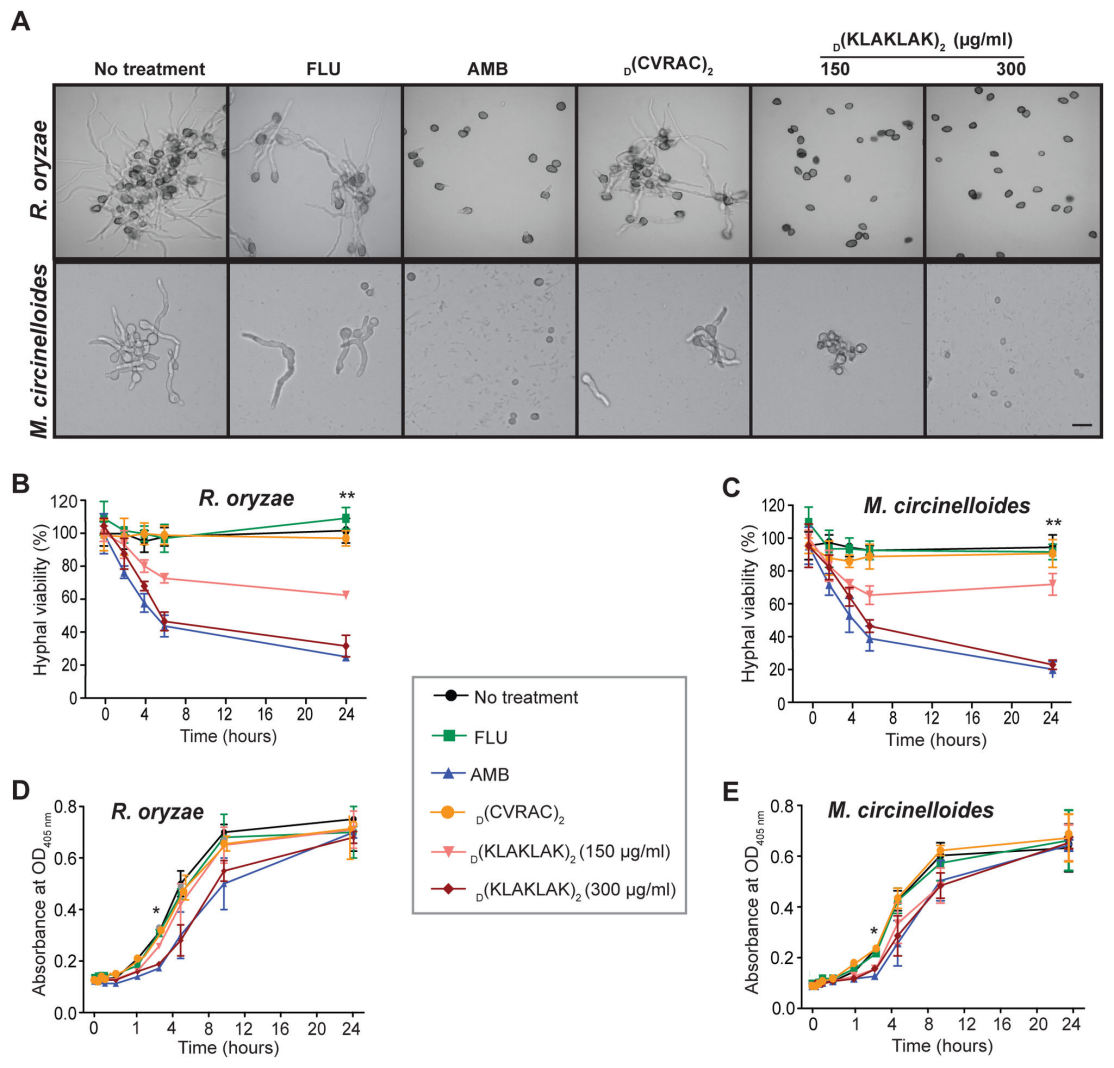
_D_(KLAKLAK)_2_ has fungicidal activity against Mucorales. (**A**) Micrographs of *R. oryzae* and *M. circinelloides* spores after six hours of exposure to AMB (4 µg/ml), FLU (128 µg/ml), _D_(KLAKLAK)_2_ (150 µg/ml, 300 µg/ml) or a negative control peptidomimetic, _D_(CVRAC)_2_ (300 µg/ml), showing that germination was completely inhibited by _D_(KLAKLAK)_2_ at MIC (300 µg/ml)and by AMB (4 µg/ml). Scale bar, 200 µm. (**B** and **C**) Mycelia were incubated with AMB (4 µg/ml), FLU (128 µg/ml), _D_(KLAKLAK)_2_ (150 µg/ml, 300 µg/ml) or _D_(CVRAC)_2_ (300 µg/ml). The extent of hyphal damage was monitored over time by the XTT reduction assay, which indicated _D_(KLAKLAK)_2_-induced dose-dependent killing relative to control drugs (*p ≤ 0.0001). (**D** and **E**) Spores were exposed to AMB (4 µg/ml), FLU (128 µg/ml), _D_(KLAKLAK)_2_ (150 µg/ml, 300 µg/ml) or the negative control peptidomimetic (300 µg/ml) for one hour. _D_(KLAKLAK)_2_ created a post-antifungal effect demonstrated by a shift of the growth curve to the right compared to the control drugs. The lag period was increased by approximately three hours (**p ≤ 0.001), followed by a rapid recovery.

To further provide proof-of-concept that _D_(KLAKLAK)_2_ is active against Mucorales, we examined its drug effect on germination. After testing a range of _D_(KLAKLAK)_2_ concentrations (18.75-300 µg/ml), we determined that germination of both *R. oryzae* and *M. circinelloides* was reduced, starting at 75 µg/ml, and with complete inhibition observed at 300 µg/ml. Notably, the activity of _D_(KLAKLAK)_2_ at 300 µg/ml concentration was similar to that of the gold-standard drug AMB in regard to germination arrest (Figure 1A). A negative control peptidomimetic _D_(CVRAC) [19] as well as fluconazole (FLU), an azole with no activity against Mucorales, had no detectable effect on the formation of germlings (Figure 1A).

Given that _D_(KLAKLAK)_2_ inhibited germination, we next examined its activity against the invasive development form of the fungus, the hyphae. We used an established 2,3-bis (2-methoxy-4-nitro-5-sulfophenyl)-5-[(phenylamino) carbonyl]-2H-tetrazolium hydroxide (XTT) reduction assay, in which the conversion of this compound to colored formazan in the presence of metabolic activity correlates with cell survival and growth [27,28]. We found a decrease by nearly 40% percent in hyphal viability at one-half MIC (150 µg/ml) of _D_(KLAKLAK)_2_ for both *R. oryzae* (Figure 1B) and *M. circinelloides* (Figure 1C). Similar to AMB, exposure to _D_(KLAKLAK)_2_ at MIC concentrations (300 µg/ml) resulted in ~70% reduction in viability relative to untreated hyphae (p ≤ 0.0001). As expected, the negative control peptidomimetic _D_(CVRAC) and FLU had no detectable effect on hyphal development.

Finally, we assessed the ability of _D_(KLAKLAK)_2_ to induce a post-antifungal effect. Spores exposure to the peptidomimetic (300 µg/ml) for one hour resulted in a three hours delay in the onset of the logarithmic growth for both *R. oryzae* (Figure 1D) and *M. circinelloides* (Figure 1E) compared to that of untreated-, FLU- or control peptidomimetic-exposed conidia (p ≤ 0.001). Collectively, these results suggest that _D_(KLAKLAK)_2_ has marked fungicidal activity against Mucorales, causing death of both spores and hyphae; however, its post-antifungal effect appears more limited.

### _D_(KLAKLAK)_2_-induced hyphal damage is the result of plasma membrane depolarization

**Figure 2:**
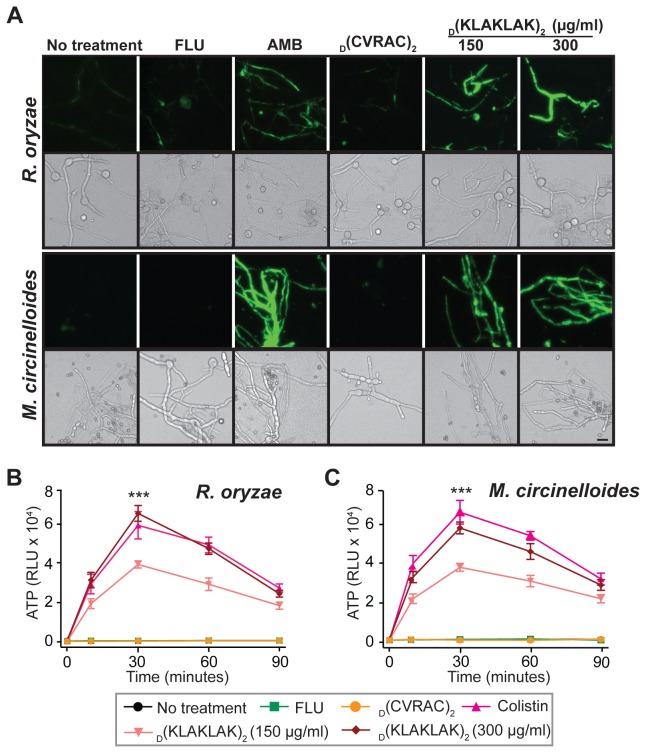
_D_(KLAKLAK)_2_ alters plasma membrane homeostasis. (**A**) Fluorescence micrographs of *R. oryzae* and M. circinelloides mycelia post-exposure to AMB (2 µg/ml), FLU (128 µg/ml), _D_(KLAKLAK)_2_ (150 µg/ml, 300 µg/ml) or _D_(CVRAC)_2_ (300 µg/ml). The DiBAC bright green fluorescence is indicative of a loss of viability due increased membrane permeability. Differential interference contrast (DIC) images served as controls for the presence of germlings. Scale bar, 200 µm. (**B** and **C**) The dose-dependent cellular ATP release in the medium after *R. oryzae* and M. circinelloides hyphae treatment with _D_(KLAKLAK)_2_ (150 µg/ml, 300 µg/ml) was similar to that induced by colistin (32 µg/ml) (***p ≤ 0.0002).

To determine whether _D_(KLAKLAK)_2_ would exert a similar mechanism of action to that observed in bacteria [23], we assessed the ability of the peptidomimetic-treated hyphae to uptake the membrane potential (ΔΨ_m_) -sensitive probe bis-(1,3-dibutylbarbituric acid) trimethine oxonol (DiBAC), which enters preferentially depolarized cells [29]. As is true for bacteria, _D_(KLAKLAK)_2_ triggered substantial dye uptake, indicating increased membrane depolarization; whereas the marker remained undetected in samples treated with negative controls such as _D_(CVRAC) or FLU (Figure 2A).

Because disruption of lipid bilayer integrity is often followed by release of chemically-stored energy from cells, we subsequently measured the ATP concentration into the media upon drug treatment [24,29]. We detected rapid (~5 min) and dose-dependent ATP release similar to that induced by colistin, an agent known to cause cell membrane disruption (p ≤ 0.0002) [24]. The efflux peaked at 30 min of incubation, followed by a slow decline for both *R. oryzae* (Figure 2B) and *M. circinelloides* (Figure 2C). These results indicate that lipid bilayer damage accompanied by ΔΨ_m_ dissipation sets in upon exposure to _D_(KLAKLAK)_2_.

### _D_(KLAKLAK)_2_ alters *R. oryzae* vacuolar homeostasis and induces mitochondrial swelling

**Figure 3:**
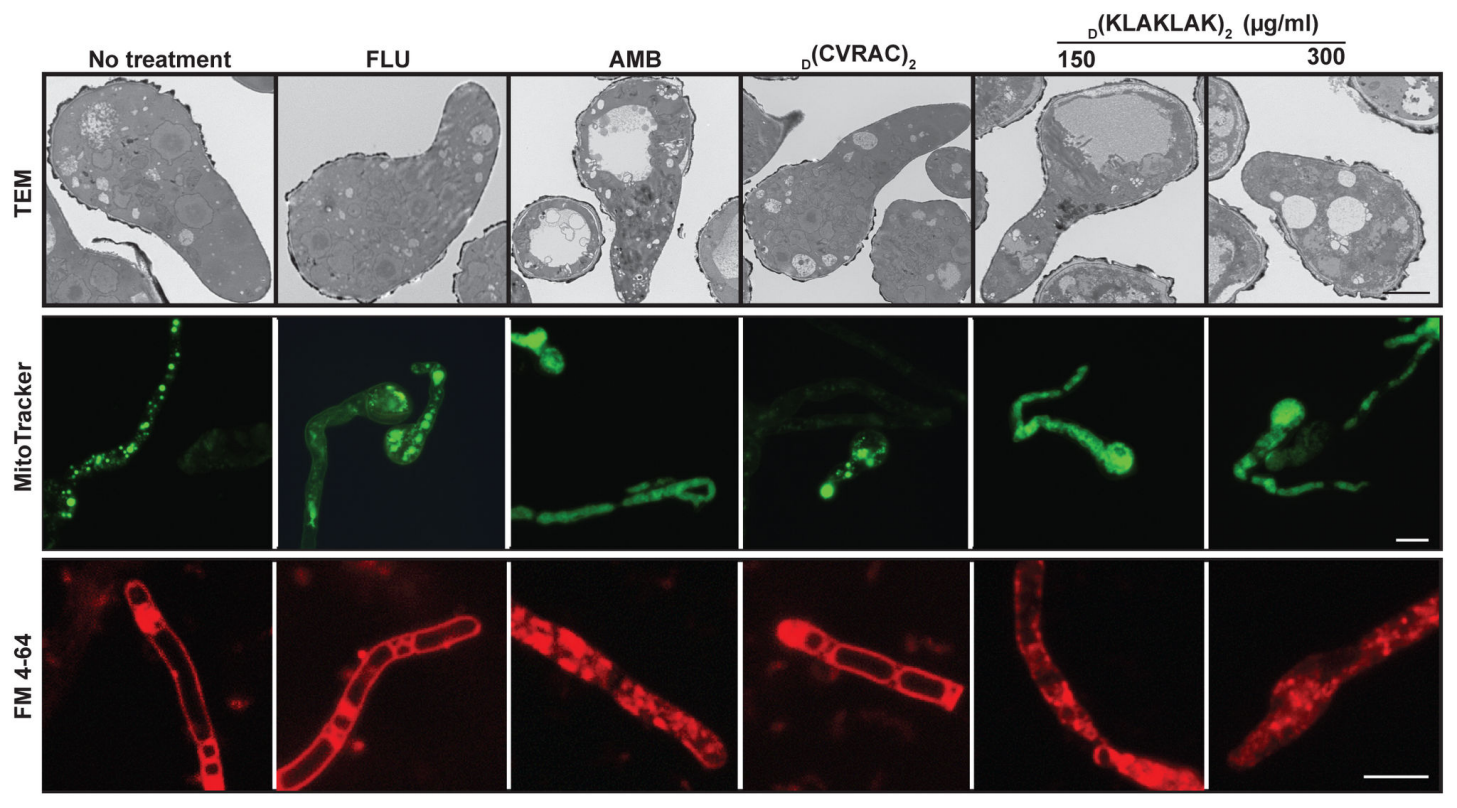
_D_(KLAKLAK)_2_ induces ultrastructural cellular changes in *R. oryzae*. TEM of germlings (6,000-fold) incubated with _D_(KLAKLAK)_2_ (150 µg/ml, 300 µg/ml) revealed extensive vacuolization comparable to that induced by AMB (2 µg/ml). The increase in number and size of vacuoles was visualized with the vacuolar probe FM4-64 (red). staining of intact plasma membrane FM4-64 diffusion into the cytoplasm and staining of fragmented vacuoles in treated _D_(KLAKLAK)_2_ (150 µg/ml, 300 µg/ml) and AMB (2 µg/ml). MitoTracker staining (green) indicated morphological changes and swelling of mitochondria in _D_(KLAKLAK)_2_-exposed germlings as opposed to the punctate pattern observed in samples treated with control drugs. TEM scale bar, 2 µm. Fluorescent micrographs scale bar, 200 µm.

To demonstrate direct plasma membrane structural damage, we used transmission electron microscopy (TEM) to observe severe cellular structural damage. In germlings incubated with either _D_(KLAKLAK)_2_ (150-300 µg/ml) or AMB (2 µg/ml), cytoplasm developed gigantic and abnormally distributed vacuoles compared to untreated, negative control peptidomimetic- or FLU-treated germlings (Figure 3). Vacuolar fragmentation is indicative of lipid bilayer damage and likely impaired trafficking.

To further assess vacuolar injury, we stained mycelia with the vital dye FM4-64, which stains plasma and vacuolar membranes [30]. In drug-free and FLU-treated controls, the marker diffused throughout the plasma membrane revealing the cell contour (Figure 3) [36]. In contrast, abundant FM4-64 diffused throughout the cytoplasm in _D_(KLAKLAK)_2_-exposed mycelia, suggesting either aggregation and fragmentation or expansion of vacuoles (Figure 3). At the same time, the membrane staining was lost likely due to lipid bilayer damage.

In previous work, we showed that upon internalization into eukaryotic cells, _D_(KLAKLAK)_2_ causes lethal mitochondrial swelling followed by caspase-3 activation and cell apoptosis [14]. Thus, we reasoned that mitochondrial injury may also be a major contributor to the fungal susceptibility. To assess mitochondrial structural changes, we performed MitoTracker Green FM staining [37], in which the probe accumulates in the organelle regardless of the ΔΨ_m_. Indeed, in vehicle- (phosphate-buffered saline, PBS), FLU- and control peptidomimetic-treated samples, the MitoTracker probe accumulated in the mitochondria and could be visualized as round, discrete structures inside the germlings. In contrast, the mitochondria of AMB and _D_(KLAKLAK)_2_-treated cells appeared to expand throughout the entire cellular content, suggesting that _D_(KLAKLAK)_2_ causes mitochondrial injury and swelling.

### _D_(KLAKLAK)_2_-induced mitochondrial injury triggers cell apoptosis

**Figure 4:**
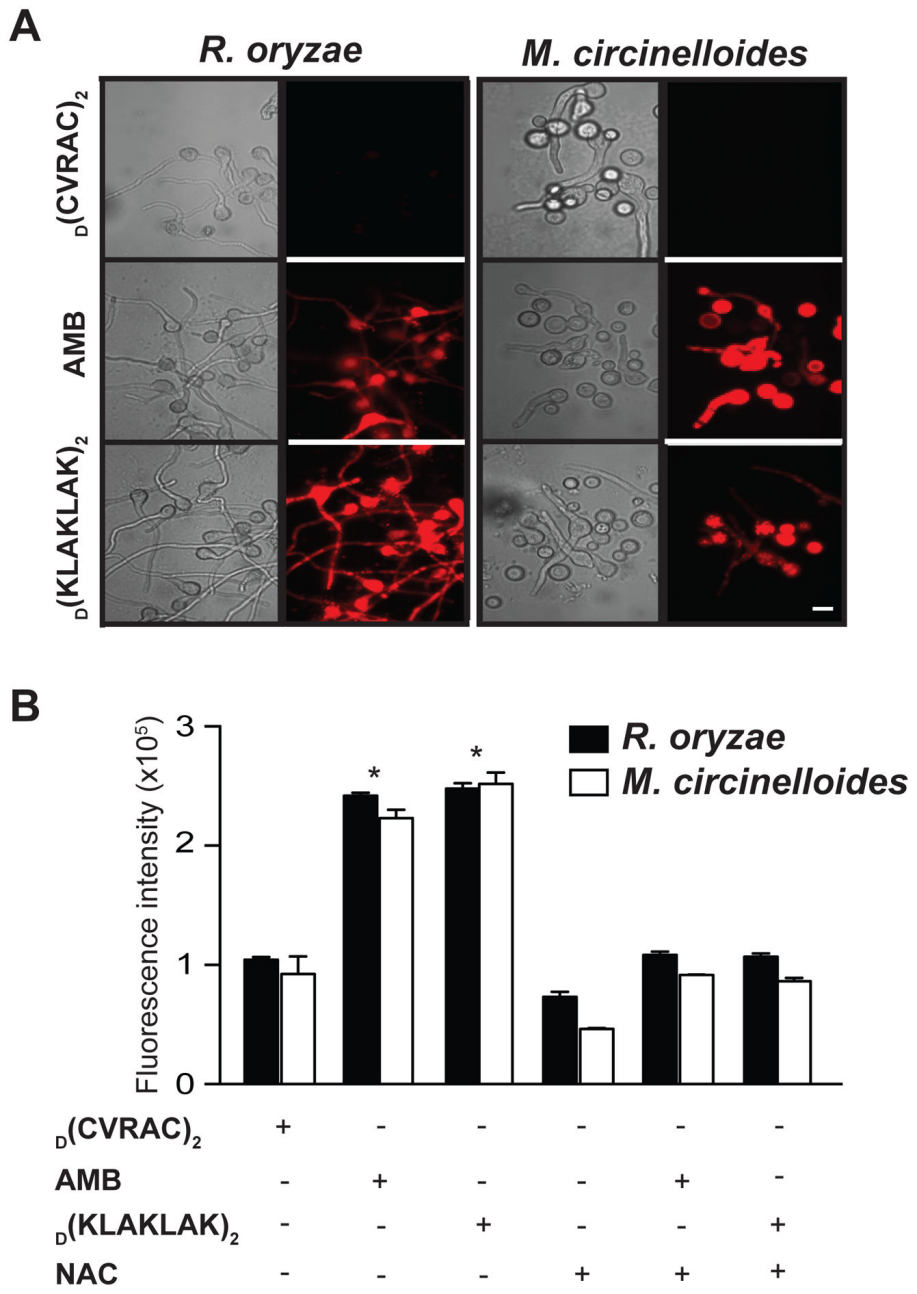
Exposure to _D_(KLAKLAK)_2_ triggers intracellular ROS accumulation. (**A**) Fluorescence and DIC micrographs of *R. oryzae* and *M. circinelloides* germlings stained with the oxidation-sensitive dye DHR-123, showing ROS production after incubation with _D_(KLAKLAK)_2_ (150 µg/ml) or AMB (2 µg/ml). Scale bar, 200 µm. (**B**) Quantitative analysis recorded with a microplate reader (excitation, 488 nm; emission, 525 nm), demonstrating a significant increase in ROS release from mitochondria in the presence of _D_(KLAKLAK)_2_ (**p ≤ 0.001) relative to FLU (128 µg/ml) or negative control peptidomimetic (300 µg/ml).

Similar to mammalian cells, augmented ROS levels [31,38], decreased mitochondrial ΔΨ_m_ [33], elevated cytoplasmic cytochrome c (cyt *c*) [39] and caspase-like activation of proteases [40,41] are hallmarks of apoptosis in molds. Because mitochondria are both a source and a target of ROS [42], we first examined ROS formation upon _D_(KLAKLAK)_2_ treatment. In both *R. oryzae* and *M. circinelloides*, germlings exposed to the peptidomimetic revealed increased intracellular ROS production as shown by the oxidation of non-fluorescent dihydrorhodamine 123 (DHR-123) to fluorescent rhodamine 123 (RH-123) [31,38] (Figure 4A). Similar to the respiratory burst triggered by AMB, quantitative analysis indicated an approximately 3-fold increase in intracellular ROS accumulation (Figure 4B) (p ≤ 0.001). In contrast, ROS levels remained unchanged when germlings were treated with the negative control peptidomimetic _D_(CVRAC) (Figure 4 A and B). N-acetyl cysteine (NAC), an ROS scavenger [31], restored normal ROS levels (Figure 4 A and B).

**Figure 5:**
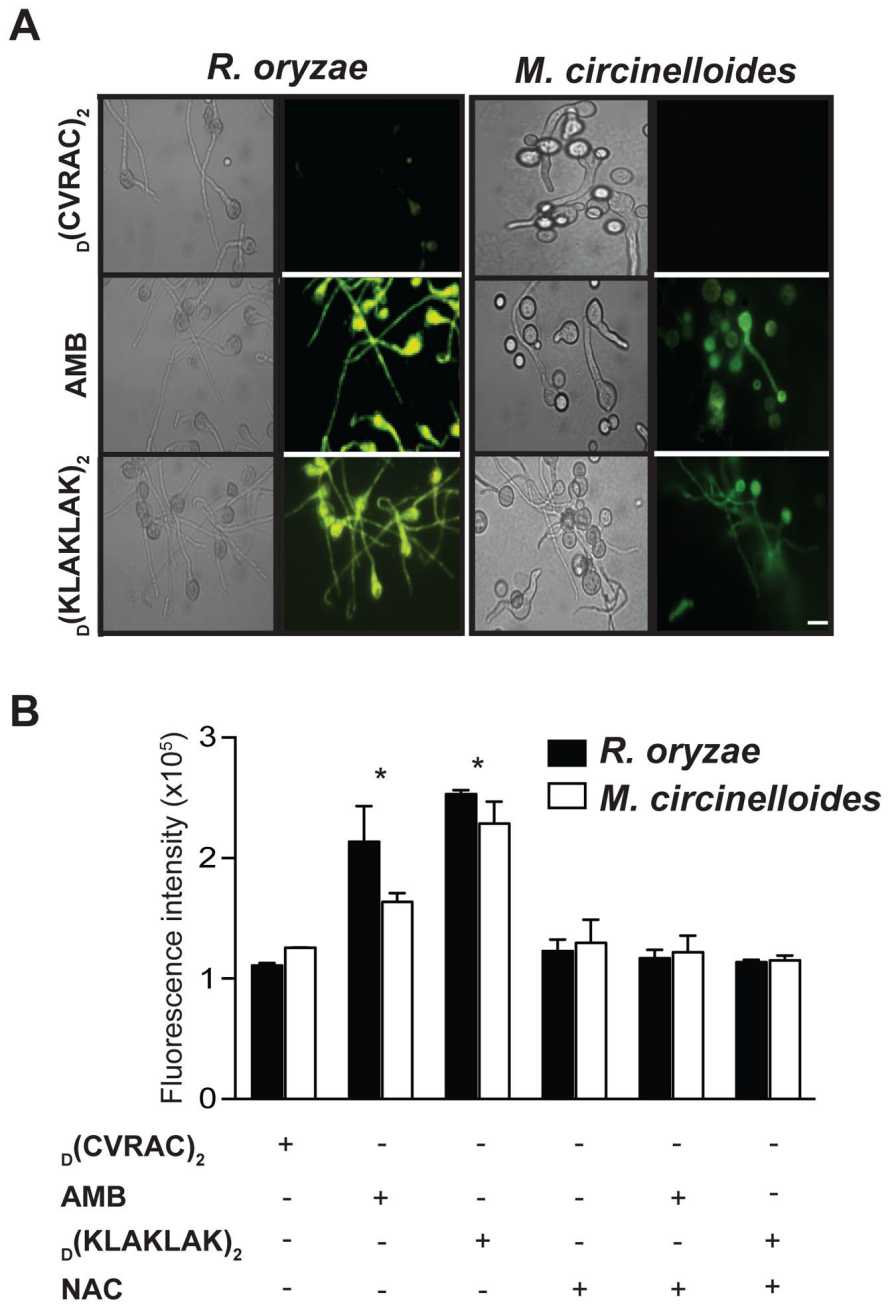
_D_(KLAKLAK)_2_ induces mitochondrial membrane depolarization (ΔΨ_m_). (**A**) Fluorescence and DIC micrographs of *R. oryzae* and *M. circinelloides* germlings stained with the oxidation-sensitive dye RH-123, indicating membrane depolarization after incubation with _D_(KLAKLAK)_2_ (150 µg/ml) or AMB (2 µg/ml). Scale bar, 200 µm. (**B**) Quantitative analysis recorded with a microplate reader (excitation, 488 nm; emission, 525 nm), demonstrating significant membrane depolarization levels (**p ≤ 0.001), post-exposure to _D_(KLAKLAK)_2_ compared to FLU (128 µg/ml) or the negative control peptidomimetic (300 µg/ml).

We also compared the mitochondrial ΔΨ_m_ of *R. oryzae* and *M. circinelloides* germlings exposed to _D_(KLAKLAK)_2_ or the negative control peptidomimetic by using RH-123, a cationic dye that distributes electrophoretically within the mitochondrial membrane as a consequence of its electric potential [33]. Consistent with our mechanistic hypothesis, _D_(KLAKLAK)_2_ treatment reduced the ΔΨ_m_ and increased membrane depolarization of both *R. oryzae* and *M. circinelloides* germlings, as demonstrated by the accumulation of RH-123 (Figure 5 A and B). The mean relative fluorescence intensity was nearly 2.4-fold higher in samples treated with _D_(KLAKLAK)_2_ compared to germlings treated with controls (Figure 5 A and B) (p ≤ 0.001). The homeostatic ΔΨ_m_ was again restored by the addition of NAC (Figure 5 A and B).

**Figure 6:**
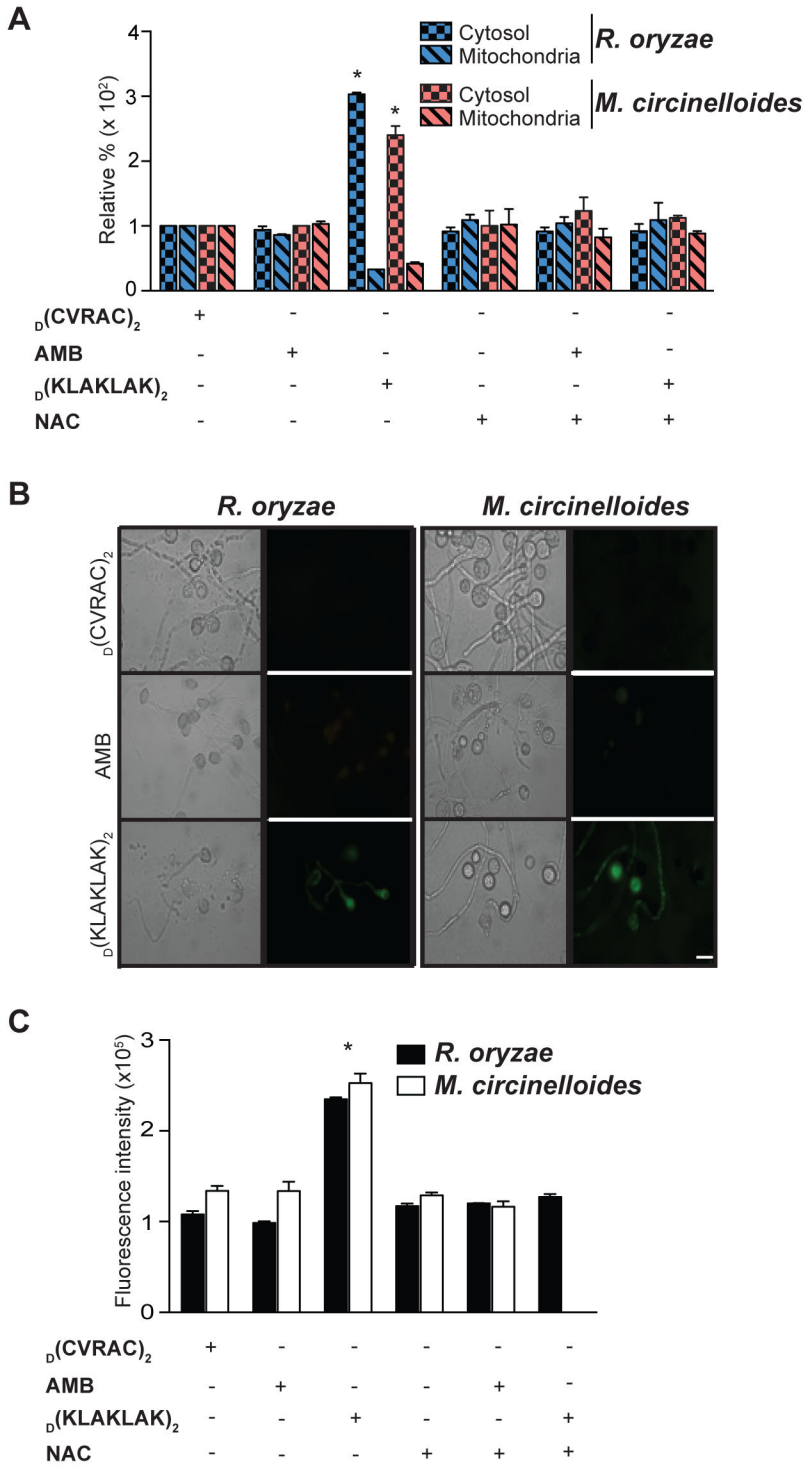
_D_(KLAKLAK)_2_ causes Mucorales apoptosis. (**A**) Relative quantification indicated significantly higher levels (**p ≤ 0.001) of cytochrome *c* release from mitochondria of *R. oryzae* and *M. circinelloides* into the cytosol in the presence of _D_(KLAKLAK)_2_ (150 µg/ml) relative to FLU (128 µg/ml) or the negative control peptidomimetic (300 µg/ml). (**B**) Fluorescence and DIC micrographs of *R. oryzae* and *M. circinelloides* germlings stained with the CaspACE FITC-VAD-FMK In Situ Marker, indicating metacaspase activation upon incubation with _D_(KLAKLAK)_2_ (150 µg/ml) compared to FLU (128 µg/ml) or the negative control peptidomimetic (300 µg/ml). Scale bar, 200 µm. (**C**) Fluorescence quantitative analysis of *R. oryzae* and *M. circinelloides* germlings in the presence of drugs revealed significant levels of metacaspase activation in germlings treated with _D_(KLAKLAK)_2_ (**p ≤ 0.001).

To further demonstrate that _D_(KLAKLAK)_2_-induced mitochondrial injury triggers apoptosis, we examined translocation of cyt *c* from mitochondrial cristae into the cytosol, which is a critical event in apoptosis [39]. We detected an approximately 3-fold increase of cyt *c* levels in the mitochondria and cytosol of *R. oryzae* and *M. circinelloides* germlings treated with _D_(KLAKLAK)_2_ relative to samples treated with the negative control peptidomimetic (Figure 6A) (p ≤ 0.001).

Similar to mammalian cells, the fungal apoptotic process is accompanied by activation of caspase-like cysteine proteases, referred to as metacaspases [40,41]. To further corroborate our results, we evaluated the activation of these enzymes in both *R. oryzae* and *M. circinelloides* germlings treated with _D_(KLAKLAK)_2_ by using an *in situ* marker, CaspACE FITC-VAD-FMK, which is a fluorescently-labeled compound that irreversibly binds to activated caspases [35]. Germlings treated with _D_(KLAKLAK)_2_ exhibited enzymatic activity as shown by fluorescent micrographs and quantitative analysis (Figure 6 B and C). In contrast, metacaspase levels remained undetected in germlings treated with the negative control peptidomimetic _D_(CVRAC). As expected, *R. oryzae* and *M. circinelloides* germlings pretreatment with the ROS scavenger NAC prevented activation of metacaspases (Figure 6 B and C). Given that caspases play a central role in the apoptotic cascade [40], these results further support the conclusion that _D_(KLAKLAK)_2_-induced mitochondrial damage elicits cell apoptosis and death. Precisely why AMB, which also affects mitochondrial membrane, does not induce apoptosis is unclear. It is possible that the antifungal mechanism of AMB activity relies mostly on plasma membrane damage, and that mitochondrial membrane damage is not as extensive; however, our methods were not as sophisticated to dissect this subtle changes. Alternatively, _D_(KLAKLAK)_2_-induced apoptotic process is only in part due to the mitochondrial membrane damage.

## Conclusions

In summary, these results show that the prototype _D_(KLAKLAK)_2_ acts as a growth proliferation inhibitor *in vitro* against Mucorales. These findings support our previous studies that this all-D-enantiomer antimicrobial peptidomimetic functions through a plasma membrane-disruptive mechanism [22,23]. Similar to other ligand-directed _D_(KLAKLAK)_2_ –containing peptidomimetics being evaluated as vascular-targeted drug candidates against cancer and obesity [14-22], untargeted _D_(KLAKLAK)_2_ enters the cell and triggers mitochondrial membrane depolarization and swelling, ultimately resulting in apoptosis and fungal death.

Mitochondria are well-known to play an important mechanistic role in the apoptotic death of *C. albicans*, *A. fumigatus*, and *A. flavus* treated with compounds such as plagiochin E or farnesol [33,42-45]. As hyperbaric oxygen potentiates the effect of antifungals [46], perhaps through accelerated induction of apoptosis [47], mitochondrial injury might indeed be the proverbial Achilles’ heel in Mucorales susceptibility. Indeed, we have recently shown that the combined inhibition of ergosterol synthesis and mitochondrial respiration results in accelerated apoptotic death [32]. One can speculate that _D_(KLAKLAK)_2_ or an optimized drug derivative may also have desirable therapeutic effects for therapy in combination with conventional antifungals.

Of note, we have previously reported a ligand peptide that binds to the cell surface of *A. fumigatus* both *in vitro* and in lung tissue in an experimental mouse model of invasive pulmonary aspergillosis [48]. Thus, we would anticipate that _D_(KLAKLAK)_2_ may perhaps be synthesized *in tandem* to Mucorales-targeting ligand motifs for potentially improved activity and decreased toxicity. In addition to favorable tissue permeability and biodistribution associated with small molecules, resistance to proteolysis may improve bioavailability and cost-effectiveness. It remains to be determined whether or not the prototype proposed here retains efficacy and is nontoxic *in vivo*, particularly in the setting of severe fungal infections in immunosuppressed patients; however toxicology studies of targeted _D_(KLAKLAK)_2_-containing drugs yielded predictable and reversible drug-induced toxicity at therapeutic concentrations [19-21]. Given the lack of good treatment options against mucormycosis, the results reported here raise the prospect for further preclinical studies to evaluate this class of antimicrobials for translational drug development.
